# Rectal carcinoma and multiple gastrointestinal stromal tumors (GIST) of the small intestine in a patient with neurofibromatosis type 1: a case report

**DOI:** 10.1186/s12957-017-1231-3

**Published:** 2017-08-23

**Authors:** Yuhei Hakozaki, Shinichi Sameshima, Teppei Tatsuoka, Takashi Okuyama, Yukinori Yamagata, Tamaki Noie, Masatoshi Oya, Akiko Fujii, Yoshihiko Ueda, Chieko Shimura, Kazumoto Katagiri

**Affiliations:** 1 Department of Surgery, Dokkyo Medical University Koshigaya Hospital, 2-1-50, Minami Koshigaya, Koshigaya, Saitama, 343-8555 Japan; 2 Department of Pathology, Dokkyo Medical University Koshigaya Hospital, 2-1-50, Minami Koshigaya, Koshigaya, Saitama, 343-8555 Japan; 3 Department of Dermatology, Dokkyo Medical University Koshigaya Hospital, 2-1-50, Minami Koshigaya, Koshigaya, Saitama, 343-8555 Japan

## Abstract

**Background:**

Neurofibromatosis type 1 (NF1) is an autosomally dominant inherited disorder characterized by multiple pigmented skin spots (*café-au-lait* spots) and neurofibroma. NF1 is associated with a wide variety of benign or malignant tumors. We report a NF1 patient who received surgical treatment for rectal carcinoma and multifocal small intestinal gastrointestinal stromal tumors (GISTs).

**Case presentation:**

A 70-year-old female patient with NF1 was referred to our hospital after a positive fecal occult blood test. Locally advanced rectal carcinoma was detected in the upper rectum using colonoscopy. A submucosal tumor 20 mm in diameter was detected in the duodenal bulb during the upper gastrointestinal endoscopy. The biopsy specimen from the duodenum was GIST with positive immunostaining of KIT and CD34 microscopically. Laparoscopic low anterior resection for rectal carcinoma and local excision of the duodenal GIST were performed successfully. During the operation, five white small nodules were found on the serosa of the jejunum. One nodule was excised for histological examination. The resected rectal tumor was a well-differentiated adenocarcinoma with multiple lymph nodes metastases according to the histology. The duodenal tumor was found to be low-risk GIST. Moreover, the nodule from the jejunum was very low risk GIST. An excised skin wart was neurofibroma according to the histology.

**Conclusions:**

GIST or carcinomas have been reported to occasionally occur in the digestive tract of the patients with NF1. We present a rare case of a NF1 patient with GISTs and colorectal carcinoma.

## Background

Neurofibromatosis type 1 (NF1), also called Von Recklinghausen disease, is an autosomal dominant disorder characterized by multiple pigmented skin spots and neurofibromas. NF1 patients are predisposed to a wide variety of benign and malignant tumors. NF1 patients are also at increased risk of getting gastrointestinal stromal tumors (GISTs).

## Case presentation

A 70-year-old female was referred to our hospital after a positive fecal occult blood test. The patient had hundreds of skin warts and pigmented spots on her body. None of her family had such skin lesions.

**Fig. 1:**
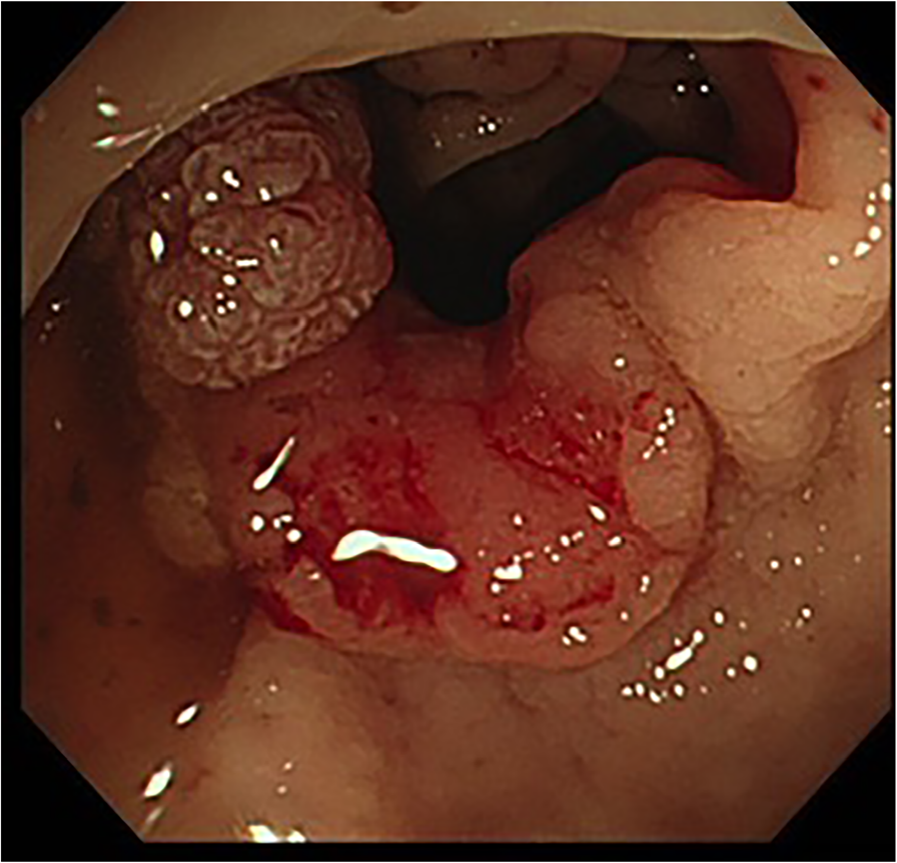
Colonoscopy showing the locally advanced rectal carcinoma

**Fig. 2:**
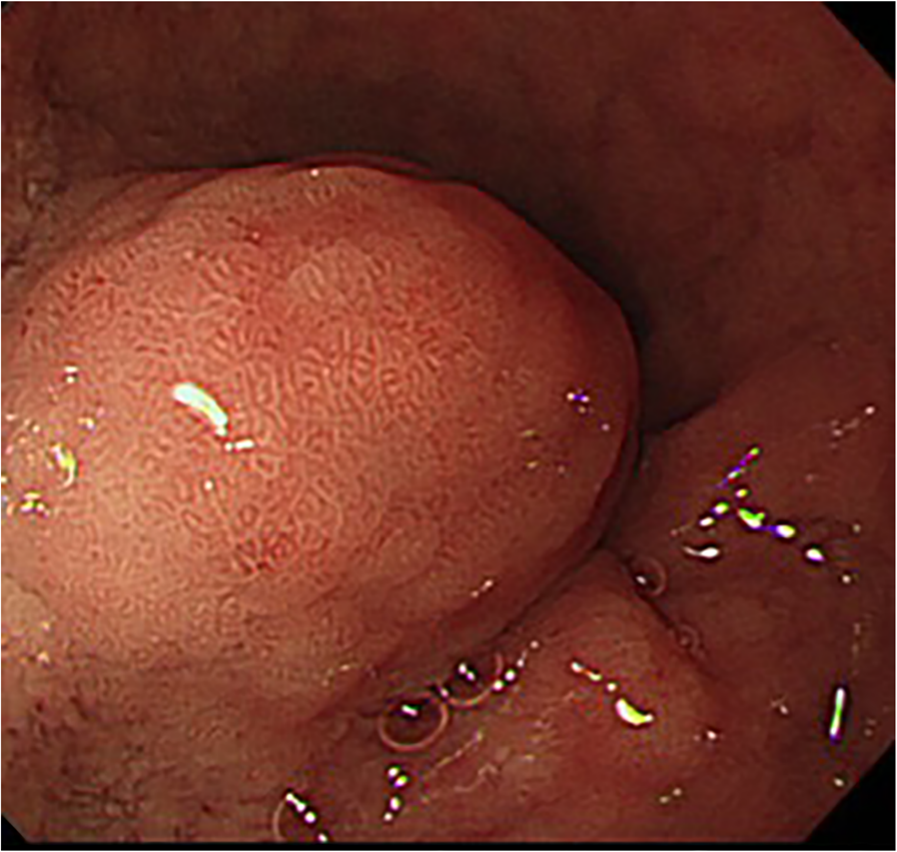
Submucosal tumor 20 mm in diameter detected in the duodenal bulb during the upper gastrointestinal endoscopy

Rectal carcinoma was detected in the upper rectum during colonoscopy (Fig. 1). Histological examination of a mucosal biopsy revealed well-differentiated adenocarcinoma. A submucosal tumor 20 mm in diameter was detected in the duodenal bulb during the preoperative screening upper gastrointestinal endoscopy (Fig. 2).

Histological examination of duodenal biopsy specimen showed spindle-shaped cells with positive immunohistochemical staining for KIT and CD34. S100 was negative for staining. The duodenal tumor was diagnosed as GIST.

**Fig. 3:**
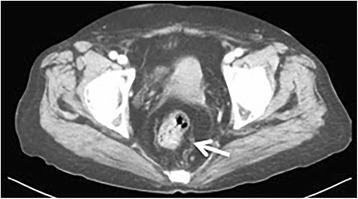
Pelvic contrast computed tomography (CT) scan showing wall thickening with contrast effect in the rectum and swelling of the lymph nodes

**Fig. 4:**
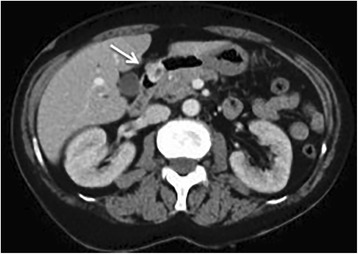
A tumor with contrast effect found (on abdominal contrast CT scan) to protrude into the duodenal cavity

Abdominal-contrast CT showed the rectum wall and swelling of the lymph nodes in the mesorectum (Fig. 3). A duodenal tumor was found to protrude into the duodenal bulb (Fig. 4). Carcinoembryonic antigen (CEA) and carbohydrate antigen 19–9 (CA19-9) levels were within normal ranges.

Surgical exploration was undertaken and a diagnosis of rectal carcinoma with duodenal GIST was made. Laparoscopic low anterior resection with lymph nodes resection for the rectal carcinoma was performed followed by local resection of the duodenum with mini-laparotomy. During the operation, five white small nodules 3–6 mm in diameter were observed on the serosal surface of the jejunum approximately 20 cm from the Treiz ligament; one 6-mm nodule was excised for histological examination. A covering ileostomy was created, and one abdominal skin wart was excised for histological examination. The patient progressed favorably post operation and was discharged.

**Fig. 5:**
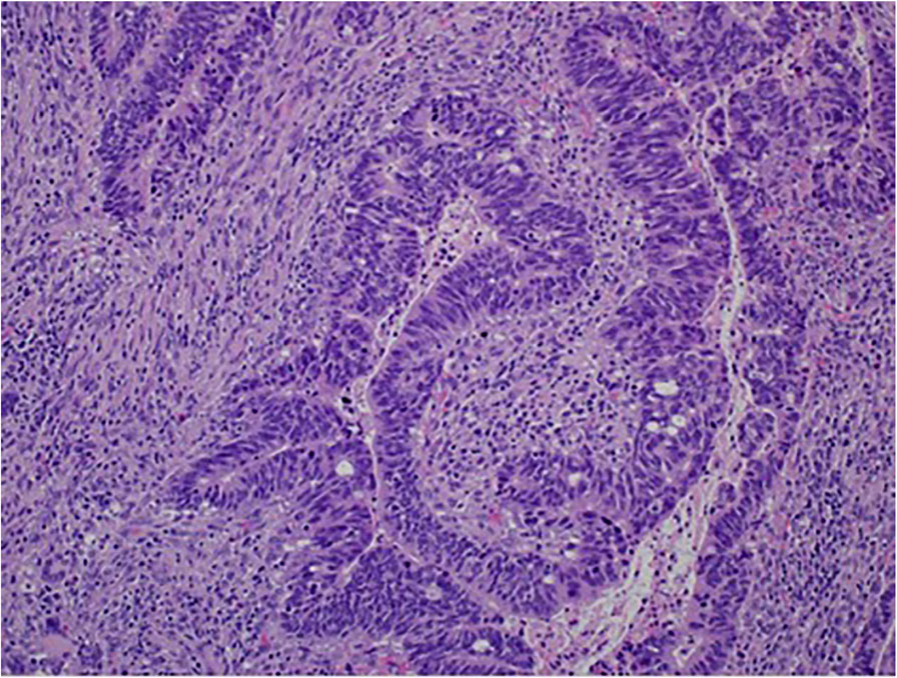
Well-differentiated adenocarcinoma of the rectum (× 200)

**Fig. 6:**
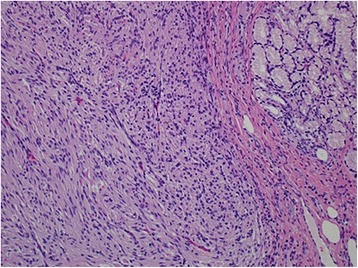
GIST of the duodenum (× 200) with 1 mitosis per 50 high-power fields (HPF). Risk of tumor progression was low

**Fig. 7:**
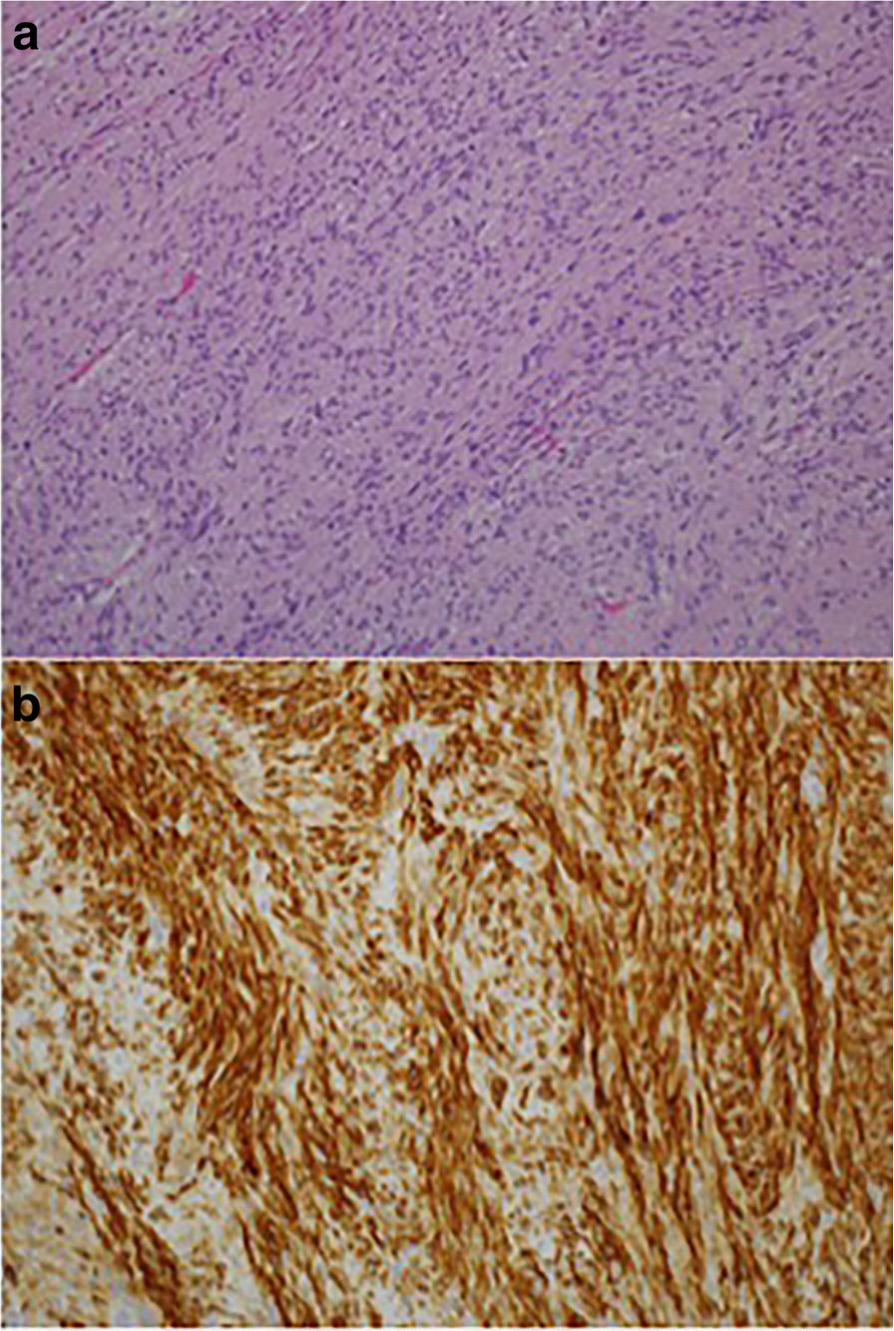
GIST of the jejunum with no mitosis per 50 HPF (× 200). **a** Risk of tumor progression was very low. **b** Positive immunostaining of KIT

**Fig. 8:**
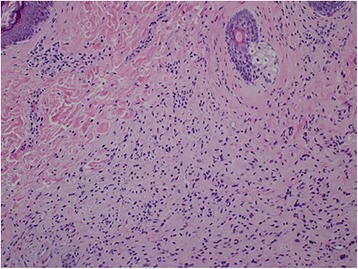
Neurofibroma of the skin (× 200)

Histologically, the resected rectal carcinoma was a well-differentiated adenocarcinoma with (four) lymph node metastasis (Fig. 5)—T2N2aM0 stage IIIB according to the TNM classification of UICC, seventh edition [1] (Fig. 7). The resected duodenal tumor was 22 mm in diameter and showed 1 mitosis per 50 high-power fields (HPF) microscopically (Fig. 6). Risk of tumor progression was low according to the modified-Fletcher classification [2]. A resected small nodule from the jejunum was also positive for KIT and CD34 immunohistochemically (Fig. 7a, b) but showed no mitosis per 50 HPF and the risk was very low. The resected skin wart was confirmed as neurofibroma after nodules showed proliferated spindle-shaped cells with unclear boundaries microscopically (Fig. 8).

The patient took adjuvant chemotherapy with capecitabine plus oxaliplatin [3]. However, it was discontinued after three courses due to severe diarrhea.

## Discussion

NF1 is an autosomal dominant disorder characterized by multiple pigmented skin spots and neurofibroma. Among the general population, its estimated incidence is 1 in 3000 births [4]. An increased incidence of nervous system and non-nervous system tumors such as neurofibromas, malignant peripheral nerve sheath tumors, and gliomas has been reported in the general population [5, 6]. The frequency of malignancy in NF1 patients was reported to be higher than that expected in the general population. The development of NF1-associated tumors is largely explained by the underlying defect of the NF1 gene [7]. The NF1 protein (neurofibromin 1) negatively regulates RAS proteins through GTPase activity [8, 9].

Agaimy described gastrointestinal manifestations of NF1 [10]. They are true neurogenic neoplasms, interstitial cell of Cajal lesions such as GIST, neuroendocrine tumors, and adenocarcinomas. Neuroendocrine tumors arising in the ampullary or periampullary lesions are the most common and characteristic gastrointestinal manifestations of NF-1.

GISTs reportedly occur occasionally in patients with NF1 [4, 11]. NF1-associated GISTs were reported as multiple generally low-grade tumors localized at the jejunum, ileum, duodenum, and stomach [12–14]. In our case, the risk of GISTs was low grade in the duodenum and very low grade in the jejunum. The co-existence of a duodenal somatostatinoma and GISTs in a patient with NF1 represents a typical and almost pathognomonic feature of NF-1 [15]. In our case, neuroendocrine neoplasms were not associated.

There have been a few reports of colorectal carcinomas arising in NF1 patients. Zoller reported that how 17 of 70 NF1 patients had developed a total of 19 malignant tumors in Sweden; 4 cases of colorectal carcinoma were included [16]. Kim also reported a case of colon carcinoma in 125 Korean NF1 patients [17]. A case of synchronous multiple colon adenocarcinomas in a patient with NF1 was also reported [18].

In our case, the NF1 patient had multiple GISTs and rectal carcinoma. Apart from a few Japanese reports, no other reports in the worldwide literature have mentioned a NF1 patient with both GIST and colorectal carcinoma. It is unclear whether this colorectal carcinoma is related to the NF1 mutation or whether it is a sporadic carcinoma. The association between NF1 and adenocarcinoma of the gastrointestinal tract is thought to be casual. Negative regulation of RAS protein by neurofibromin 1 may affect the carcinogenesis of the rectal carcinoma [19]. Li reported that the germline mutations in NF1 that cause NF1 can also occur in somatic cells and contribute to the cancer development [20].

In this case, the tumor progression risks of GISTs were low in the duodenum and very low in the jejunum. Unresected small nodules of the jejunum will not affect the prognosis of the patient. The resected rectal carcinoma was locally advanced (stage IIIB). The prognosis of this patient is dependent on the relapse of the rectal carcinoma and a scheduled examination to evaluate any recurrence is required.

## Conclusions

In summary we presented a rare case of NF1 patient associated with multiple GISTs and rectal carcinoma.
